# An In-Depth Look at Nutrition Support and Adequacy for Critically Ill Children with Organ Dysfunction

**DOI:** 10.3390/children11060709

**Published:** 2024-06-08

**Authors:** Nicole Knebusch, Paola Hong-Zhu, Marwa Mansour, Jennifer N. Daughtry, Thomas P. Fogarty, Fernando Stein, Jorge A. Coss-Bu

**Affiliations:** 1 Division of Critical Care Medicine, Department of Pediatrics, Baylor College of Medicine, Houston, TX 77030, USA; 2 Texas Children’s Hospital, Houston, TX 77030, USA; 3 Department of Pediatrics, Texas Tech University Health Sciences Center, Lubbock, TX 79430, USA; 4 Department of Clinical Nutrition Services, Texas Children’s Hospital, Houston, TX 77030, USA

**Keywords:** pediatrics, nutrition support, organ dysfunction, critically ill, pSOFA score, intensive care

## Abstract

Patients admitted to a pediatric intensive care unit (PICU) need individualized nutrition support that is tailored to their particular disease severity, nutritional status, and therapeutic interventions. We aim to evaluate how calories and proteins are provided during the first seven days of hospitalization for children in critical condition with organ dysfunction (OD). A single-center retrospective cohort study of children aged 2–18 years, mechanically ventilated > 48 h, and admitted > 7 days to a PICU from 2016 to 2017 was carried out. Nutrition support included enteral and parenteral nutrition. We calculated scores for the Pediatric Sequential Organ Failure Assessment (pSOFA) on days 1 and 3 of admission, with OD defined as a score > 5. Of 4199 patient admissions, 164 children were included. The prevalence of OD for days 1 and 3 was 79.3% and 78.7%, respectively. On day 3, when pSOFA scores trended upward, decreased, or remained unchanged, median (IQR) caloric intake was 0 (0–15), 9.2 (0–25), and 22 (1–43) kcal/kg/day, respectively (*p* = 0.0032); when pSOFA scores trended upward, decreased, or remained unchanged, protein intake was 0 (0–0.64), 0.44 (0–1.25), and 0.66 (0.04–1.67) g/kg/day, respectively (*p* = 0.0023). Organ dysfunction was prevalent through the first 72 h of a PICU stay. When the pSOFA scores trended downward or remained unchanged, caloric and protein intakes were higher than those that trended upward.

## 1. Introduction

There is no doubt that nutrition support for children in a pediatric intensive care unit (PICU) has a noteworthy role to play, as the injury-induced metabolic response consists of a dysregulation of energy metabolism and a high level of protein catabolism, leading to the deterioration of lean body mass in children [1,2,3]. Nutrition support in this vulnerable population requires continued monitoring, as children need a continuous supply of calories and protein to maintain growth [1]. Critically ill children are characterized by either hemodynamic instability, the need for respiratory support, accompanied by severe comorbidities, or the need for antimicrobials to treat sepsis and infection [4]. While measuring the basal metabolic rate in children represents a challenge, the Society of Critical Care Medicine (SCCM) and the American Society of Parenteral and Enteral Nutrition (ASPEN) latest guidelines recommend that energy-estimating equations, such as Schoefield’s equation without the consideration of stress factors, should be used to estimate caloric needs when indirect calorimetry is not available. SCCM and ASPEN also recommend providing at least 1.5 gr/kg/day of protein to critically ill children to diminish negative nitrogen balances [5].

Considering that this is a vulnerable population, it is critical to focus on nutrition therapy, since malnutrition and obesity, as well as a worsening nutritional status, have been connected with detrimental clinical outcomes, including prolonged mechanical ventilation time, a longer hospital length of stay (LOS), and an increased risk of infection during a PICU stay [5,6,7]. The literature supports evidence that failing to deliver at least 66% of the daily protein and caloric requirements to children within the first seven days of a PICU stay are associated with a greater mortality risk [8,9]. Because of the nature of critical illness and stress responses, PICU patients are at risk of a worsening nutritional status throughout their hospitalization period [10].

Accompanying the increased risks associated with malnutrition, malnourishment has also been associated with an increase in pediatric sequential organ failure assessment (pSOFA) scores ≥ 5, which indicates organ dysfunction [11,12]. A pSOFA score ≥ 5 has been associated with prolonged mechanical ventilation, longer PICU and hospital stays for children after lung transplants, and increased mortality after congenital heart surgery [12,13].

Providing enteral nutrition in PICU patients, when a child is hemodynamically stable and the enteral route is available, remains a controversial topic, mainly when vasoactive-inotropic medications are used [14,15]. Studies suggest that the provision of enteral support is safe in critically ill children and adults without increased gastrointestinal symptoms [14,15]. Children who are critically ill have decreased intestinal barrier integrity, and these changes can affect and modify the development of organ dysfunction, for which enteral nutrition is recommended [5,16]. Studies evaluating enteral nutrition in the initial days of a PICU stay have shown that early enteral nutrition has a range of benefits, including shorter PICU and hospital stays, additional days without mechanical ventilation, and less organ dysfunction [17]; a decrease in mortality has been observed in critically ill children who achieve 60% adequacy of enteral calories and protein in the last decade [8,9].

This study aims to evaluate the nutrition support adequacy of calories and protein during the first week of a PICU stay in moderately to severely ill children with organ dysfunction admitted to a PICU. We hypothesize that achieving the goal adequacy of ≥60% of total calories and protein is achievable within seven days of admission when combined enteral and parenteral nutrition support is utilized. Additionally, we evaluated the nutrition support adequacy of enteral calories and protein in children receiving vasoactive–inotropic support.

## 2. Materials and Methods

### 2.1. Study Design

We performed a single-center retrospective cohort study using the database and electronic medical record (EMR) of children admitted to the Texas Children’s Hospital Pediatric Intensive Care Unit (PICU) in Houston, Texas, USA, from January 2016 to December 2017. The Baylor College of Medicine Institutional Review Board approved the study, H-39403, on 17 March 2021. This study gathered extensive data, including demographic and anthropometric measurements (weight in kilograms and height in cm) upon admission, primary diagnosis, time spent on mechanical ventilation (MV), and length of stay in a PICU and at the hospital. The Pediatric Index of Mortality (PIM2) and risk of mortality (ROM%) were calculated upon admission and the Pediatric Sequential Organ Failure Assessment (pSOFA) score and vasoactive–inotropic score (VIS) were calculated on days 1 and 3 for both.

### 2.2. Patients

Patients > 1 month and < 18 years old with a PICU admission (index admission) were included. Of 4199 PICU admissions during the study period, we identified a total of 1283 children who had a PICU stay of more than 3 days. For the nutrition support cohort (*n* = 164), we included children 2–18 years old who were mechanically ventilated for more than 48 h, were admitted to a PICU for more than 7 days and received feedings via an enteral or parenteral route. Children were excluded if mechanical ventilation started after the first seven days of admission to a PICU and the main intake was primarily by mouth.

### 2.3. Organ Dysfunction and Vasoactive–Inotropic Score

The Pediatric Sequential Organ Failure Assessment (pSOFA) score was calculated based on data obtained from medical records on days one and three of admission to a PICU for all patients included in the cohort. The pSOFA score includes respiratory, coagulation, hepatic, cardiovascular, neurological, and renal variables. The pSOFA score was defined as high if the pSOFA was ≥ 5 [11]. On day 3, children were classified according to whether their pSOFA score had increased, decreased, or remained the same. Additionally, the vasoactive–inotropic score (VIS) was obtained after 24 and 72 h of a PICU stay for the patients in the nutrition cohort using data obtained from the electronic medical record, the VIS was calculated by collecting the highest dose of vasoactive and inotrope medications during those days [18]. A VIS was defined as high if the VIS was ≥ 5. Patients were categorized on day 3 if the VIS was trending upward, trending downward, or unchanged.

### 2.4. Nutrition Support

Data on nutritional support were collected from medical records, including nutritional status, nutritional plans formulated by dietitians, and daily intakes of enteral and parenteral nutrition. Each nutritional plan was adjusted based on the criteria of a dietitian based on a patient’s clinical condition. A dietitian’s prescription included different methodologies, such as the basal metabolic rate with or without added stress factors, dietary reference intake for age, and protein intake based on guideline recommendations [19]. We evaluated nutritional status based on the Centers for Disease Control and Prevention (CDC) growth charts [20]. The prevalence of acute malnutrition (≤−2 z-score), normal nutritional status (≥−2–≤+2 z-score), and obesity (≥+2 z-score) in children was estimated via body mass index (BMI) for the age z-score (BMI/A) [21]. Nutrition support intake was calculated for all children who received parenteral or enteral nutrition. Propofol was included in the calculation of caloric intake if utilized for sedation. According to ASPEN recommendations, the basal metabolic rate was calculated using the Schoefield equation to estimate the daily caloric requirement, and the protein goal was defined as 1.5 g per kilogram per day [5]. We calculated the adequacy for calories and protein based on BMR and 1.5 g/kg/day. Nutrition adequacy was defined as [intake/recommended prescription) × 100], and goal adequacy was defined as ≥ 60% based on the prescription. Additionally, the adequacy from a dietitian’s prescription for calories and protein was calculated as [(intake/dietitians’ prescription) × 100].

### 2.5. Statistical Analysis

Statistical analyses of descriptive data are presented as means with standard deviation (SD) or medians with interquartile range (IQR, 25–75th) for continuous variables and frequencies with percentages for categorical variables. The nonparametric data were analyzed with the Mann–Whitney and Fisher exact tests, to compare continuous and categorical variables, respectively. The paired *t*-test was used to compare the same group against a reference value. The Kruskall–Wallis test was used to compare more than 2 groups. Statistical significance was established a priori *p* < 0.05. Statistical analysis was performed with Stat View Version 5.0.1 (SAS Institute Inc., Cary, NC, USA).

## 3. Results

Of the 4199 PICU admissions during the study period, we identified a total of 1283 children who had a PICU stay of more than 3 days, and 43% were female. For the total cohort of patients, the median and interquartile (IQR) for age was 11.7 (7.7–15) years, for weight it was 36.8 (23–55) kg, for the Pediatric Index of Mortality (PIM 2) risk of mortality (ROM)it was 3.71 (2.8–6.4)%, for MV duration it was 223 (157–392) h, for PICU LOS it was 12 (9.3–20) days, for hospital LOS it was 30 (19–53) days, and for hospital mortality it was 12.2%. The prevalence of normal BMI, acute malnutrition, and obesity was 75.9%, 10.5%, and 13.6%, respectively (Table 1).

A comparison of clinical variables was made according to the nutritional diagnosis of all PICU patients at the time of admission. Most children with acute malnutrition (*n* = 136) were younger [1.22 (0.62–10.4) years], and children with obesity were older (*n* = 176) [3.5 (0.83–11.1) years], while children with a normal nutritional status (*n* = 971) had an age of [2.86 (0.72–10.24) years], (*p* = 0.0052). No statistically significant comparisons were found between MV hours (*p* = 0.7047), PICU LOS (*p* = 0.4931), hospital LOS (*p* = 0.6191), PIM2 ROM (*p* = 0.8331), the pSOFA score on day 1 (*p* = 0.6791), mortality (*p* = 0.1799), and the nutritional status of the children.

The prevalence of organ dysfunction was 79.3% for day 1 and 78.7% for day 3. When comparing clinical variables according to organ dysfunction, in children without organ dysfunction (*n* = 440) the PIM2 ROM% was 1.03 (0.76–3.56)%, compared to 3.53 (1.04–5.16)% in patients with organ dysfunction (*n* = 843) (*p* < 0.0001). Out of 89 in-hospital deaths, 92.1% (*n* = 82) had organ dysfunction upon admission, compared to 7.9% (*n* = 7) without organ dysfunction (*p* < 0.001). PICU LOS was longer in patients with organ dysfunction, with a stay of 7.5 (4.7–12.6) days, compared to 5.8 (4–9) days in patients without organ dysfunction (*p* < 0.001). Likewise, children without organ dysfunction had more ventilator-free days (*p* = 0.0338) and longer hospital LOS (*p* = 0.0038). There were no statistically significant differences between age and the presence of organ dysfunction (*p* = 0.1455)

### 3.1. Nutrition Support Cohort

Out of 1283 children, 164 met the nutrition support criteria. Patient characteristics are described in Table 2. The primary diagnoses for the nutrition support cohort included respiratory disease and failure (15.9%), hematology and oncology disorders (22.6%), renal diseases (1.2%), genetic diseases (9.1%), infectious diseases (9.1%), gastrointestinal diseases (3%), trauma and surgical patients (15.9%), critical neurological illnesses (22.6%), and cardiovascular diseases (0.6%).

**Figure 1:**
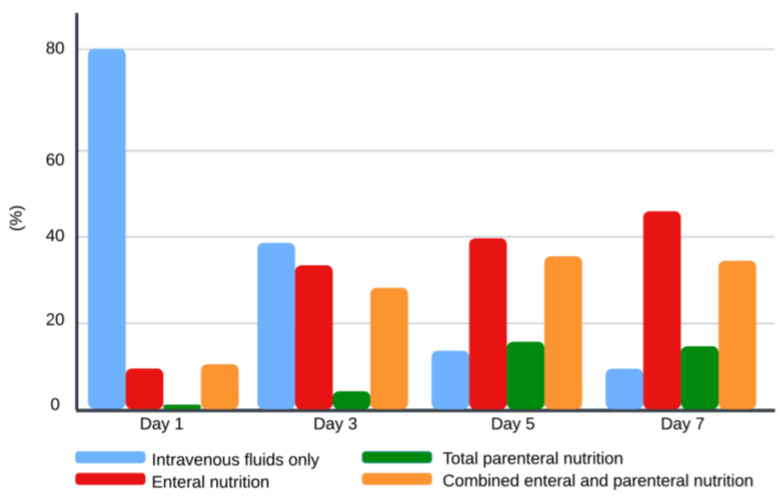
Type of nutrition support during the first week of admission. Values for each bar represent percentages for each type of nutrition support for each day.

The type of nutritional support is illustrated in Figure 1. On day 1, 80% of the children received intravenous (IV) fluids only, 9% enteral nutrition (EN), 1% parenteral nutrition (PN), and 10% combined enteral and parenteral nutrition (EN + PN). On day 3, 37% of the children were on IV fluids, 32% on EN, 4% on PN, and 27% on EN + PN. By day 5, the percentage of children receiving IV fluids decreased to 13%, 38% of children had EN, 15% of children had PN, and 34% had EN + PN. On day 7, 9% of children remained on IV fluids, 44% on EN, 14% on PN, and 33% on EN + PN. Out of 13 malnourished children, approximately one-third (*n* = 4) required PN support during the first seven days of admission.

The prescriptions from dietitians for calories were on average 44 ± 17 (SD) kcal/kg/day and 1.70 ± 0.42 g/kg/day for protein. Compared to ASPEN recommendations, protein prescription on average was appropriate [5]. We found a statistically significant distinction between the total and enteral intake for calories and protein and a dietitian’s prescription during the first seven days of admission (*p* < 0.001) (Table 3). More than half of children reached goal adequacy by day 5 when EN + PN was used. For the analysis of enteral caloric and protein adequacy, the > 60% goal was not reached during the first week of admission. Throughout the initial week of admission, the percentage of patients that reached goal adequacy for total calories for days 1, 3, 5, and 7 was 9.1%, 26%, 53%, and 62%, respectively. The percentage of patients that reached 1.5 g/kg/day of total protein intake on days 1, 3, 5, and 7 was 3.7%, 15%, 39%, and 45%, respectively. By day 3 of admission to a PICU, 72% of the patients had received enteral nutrition support, 32% were fed only by the enteral route, and 40% received EN + PN.

### 3.2. Nutrition Support Cohort

**Figure 2:**
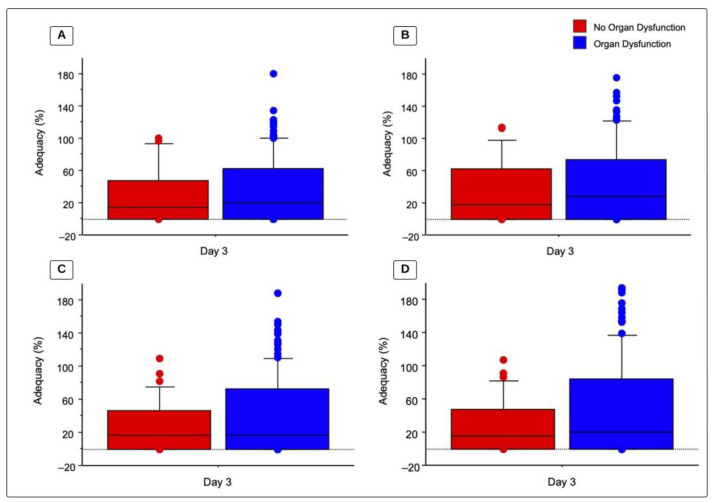
Total caloric and protein adequacy based on organ dysfunction. Values represent adequacy (%) on day 3 of admission to a PICU for patients with organ dysfunction compared to children without organ dysfunction, analysis via a Mann-Whitney U test. Box represents median and 25th–75th percentiles and whiskers and bullets represent maximum value. (**A**) Total caloric adequacy based on dietitians’ prescriptions (*p* = 0.593); (**B**) total caloric adequacy based on the recommended intake (BMR) (*p* = 0.6074); (**C**) total protein adequacy based on dietitians’ prescriptions (*p* = 0.4102); and (**D**) total protein adequacy based on the recommended intake (1.5 g/kg/day) (*p* = 0.4455).

There were no statistically significant differences between children with organ dysfunction (OD) and those without OD regarding caloric and protein intake (Table 4). Figure 2 illustrates the adequacy of caloric and protein prescriptions by dietitians and by the ASPEN recommended goal as well as OD status on day 3. Children without OD by day 3 had a total recommended caloric adequacy (BMR) of 17.9 (0–62.4)%, compared to 27.8 (0–74.4)% in children with OD (*p* = 0.6074). Children without OD by day 3 had total caloric adequacy based on dietitian prescriptions of 14.7 (0–47.3)% compared to 20.1 (0–62.4)% in children with OD (*p* = 0.5931). For protein adequacy, based on 1.5 g/kg/day, children without OD had an adequacy of 15.9 (0–48.1)%, compared to 20.1 (0–84.4)% in children with OD (*p* = 0.4455). Lastly, protein adequacy based on a dietitian’s prescription for children without OD was 16.2 (0–46.3)%, compared to 16.2 (0–73.1) in children with OD (*p* = 0.4102). By day 3 of PICU admission, 34.1% of children with OD reached recommended caloric goals of >60% BMR vs. 28.6% without OD (*p* = 0.53). Out of the 129 children with OD on day 3, 17.8% reached the protein goal of 1.5 g/kg/day compared to 2.9% of children without OD by day 3 of PICU admission (*p* = 0.0262).

### 3.3. pSOFA Score Trend and Nutrition Support

**Figure 3:**
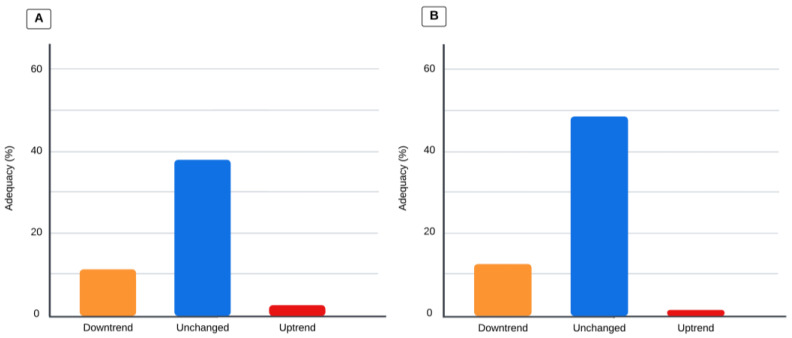
Values represent the percentage of adequacy for calories and protein. Comparison analysis via the Kruskal–Wallis test; *p*-value is significant if < 0.0167 for the three-group comparison. (**A**) Total caloric adequacy based on dietitians’ prescriptions and changes in pSOFA score trends (*p* < 0.005). (**B**) Total protein adequacy based on dietitians’ prescriptions and pSOFA score trends (*p* < 0.005).

Children who had a pSOFA score that trended upward on day 3 had a total caloric intake of 0 (0–15.0) kcal/kg/day, compared to 9.2 (0–25.3) kcal/kg/day when the pSOFA score trended downward, and 22 (1.03–42.5) kcal/kg/day when the trend remained unchanged (*p* = 0.0032). When organ dysfunction trended up via the pSOFA score on day 3, the total protein intake was 0 (0–0.64) g/kg/day, compared to 0.44 (0–1.25) g/kg/day when the trend went downward, and 0.66 (0.04–1.67) g/kg/day when the trend remained unchanged (*p* = 0.0023). Adequacy according to organ dysfunction is illustrated in Figure 3. When the pSOFA score trended upward, decreased, or remained unchanged, total caloric adequacy was 2.61 (0–25.1)%, 11.3 (0–38.9)%, and 37.9 (4.05–74.8)%, (*p* = 0.022), respectively; when the pSOFA score trended upward, decreased, or remained unchanged, total protein adequacy was 1.45 (0–28.1)%, 12.6 (0–41.7)%, and 48.5 (3.32–86.9)% (*p* = 0.015), respectively.

### 3.4. VIS and Enteral Nutrition Support

On day 3, patients with a high VIS had a lower enteral caloric adequacy of 0 (0–0)%, compared to patients with a low VIS of 0 (0–29)% (*p* = 0.0026); the enteral protein adequacy showed similar results, with adequacy of 0 (0–0)% when patients had a high VIS compared to 0 (0–23)% when the VIS was < 5 (*p* = 0.0022). Changes in enteral caloric and protein adequacy are shown in Table 5.

## 4. Discussion

Across our study, we found that the hospital mortality of our cohort of children was three to four times higher than the 2.4% mortality rate reported for all children admitted to PICUs in the United States [22,23,24]. The patients in the nutrition cohort were moderately to severely ill children, with longer PICU LOSs than the reported average stay for critically ill children in the USA [25]. The prevalence of organ dysfunction on day 1 was notoriously high (79.3%) and stayed high (78.7%) on day 3; our reported prevalence of OD compared to other reports was significantly higher than published studies with a prevalence of 12–14%, and up to 57% depending on the definition of organ dysfunction utilized and the study population [26,27]. There is an association between the number of organs involved and increased mortality of approximately 50% if multiple organs are involved [26,27]. As evidenced by this cohort, mortality was higher, with 89 (6.94%) and 20 (12.2%) deaths during their hospital stay for the two cohorts of patients included in our study.

In general, over 50% of the patients reached 60% goal adequacy by day 5 of admission to ta PICU for calories and protein, and more than 60% of the patients reached goal adequacy by day 7. These results highlight the efficacy of our nutrition support during the first week of admission, reaching the accepted target of adequacies in critically ill children [5,8,9,28]. The associations found by Bechard et al., in a study performed on 17 PICUs with 1844 children enrolled, demonstrated the association between reaching 60% goal adequacy during the first week of admission to a PICU and mortality [29]. Children in our study received parenteral nutrition combined with enteral nutrition to reach higher adequacies during the first week; results from Bechard et al. showed that the association with lower mortality was still present when enteral and parenteral nutrition was used to reach goal adequacy [29].

Our study showed that, by day 5, patients’ caloric intake based on a dietitian’s prescription indicated that 25% of the patients had received up to 100% of caloric goal adequacy; nonetheless, when adequacy is compared to BMR estimated energy needs, more than 25% of patients received over 120% of estimated caloric requirements. It is possible that some of the dietitians used stress factors for estimating caloric needs, given the fact that the BMR adequacy was higher than the prescribed adequacy; this is not in line with current SCCM and ASPEN guideline recommendations from 2017. Our nutrition support cohort followed the recommendation of avoiding stress factors to decrease the risk of overfeeding [5,30,31].

Around 50% of our cohort needed parenteral nutrition during the first seven days of admission, with the rates of parenteral nutrition use, either total or supplementary to enteral nutrition, increasing from 14% on day 1 to 47% on day 7 (14% TPN only + 33% combined PN + EN). The PEPaNIC study, a multicenter randomized controlled study, assessed the utilization of early (less than 24 h after admission) vs. late (after 7 days of admission) parenteral nutrition in critically ill children and deduced that the provision of early parenteral nutrition was associated with a higher rate of infections and prolonged hospital as well as intensive care unit stays when compared to the initiation of late parenteral nutrition [32]. Our study reflects data collected several years after the PEPaNIC study, highlighting the continued use of parenteral nutrition as an aid when enteral nutrition does not provide the recommended nutrition intake and the need to individualize nutrition support to reach caloric and protein goals [29]. A randomized control trial of 140 critically ill children by Saleh, N., et al. has evidenced that the provision of early parenteral nutrition allows reaching total adequacy faster than when children are provided enteral nutrition only [33]; therefore, the use of parenteral nutrition after the first 24 h and before day 7 of admission to a PICU needs further investigation.

When looking at the nutrition support of children with organ dysfunction vs. children without organ dysfunction, although not statistically significant, patients with organ dysfunction reached higher adequacies for calories on day 3, but similar adequacies for protein (Figure 2). Regarding the suggested protein intake of 1.5 g/kg/day, in our study almost 1 out of 5 patients with organ dysfunction reached that goal compared to less than 5% of patients without organ dysfunction. A sufficient protein intake in children who are in critical condition has been proven to mitigate a negative nitrogen balance, helping maintain enough protein provision for acute-phase proteins and immune components, while helping maintain fat-free mass [2,34,35].

While there are no published studies that evaluate the association of nutrition adequacy with organ dysfunction in critically ill patients, the provision of enteral calories and protein is thought to slow the progression of organ dysfunction by attenuating inflammatory responses, maintaining muscle mass, and regulating metabolic changes, waiting for enteral nutrition to be initiated to provide support to the intestinal barrier and microbiome [36]. Nutrition support can become a challenge in a PICU for multiple reasons, including changes in medical stability and severity, changes in mechanical ventilation, procedures, perceived gastrointestinal intolerance, medications, fluid restrictions, limited access to either enteral or parenteral nutrition, and changes in glucose [37]. We observed a significant difference in caloric and protein adequacy for days 1–3 of admission and changes in pSOFA score trends. Our study showed that when patients kept the same score of organ dysfunction from days 1 to 3, caloric and protein adequacy was higher compared to children with an uptrend in the pSOFA score. It is possible that adequacy was lowest in children with an uptrending pSOFA score because of medical instability or other barriers to providing adequate nutrition.

The provision of early enteral nutrition has been connected with lower mortality risk in multiple studies on children in critical condition [9,17,29,38]. One study by Mikhailov et al. enrolled 5105 mechanically ventilated children from 12 PICUs and demonstrated that early enteral nutrition decreased the odds of death and was less likely to be used in a group of very ill children, similar to our study, where severely ill children were less likely to receive enteral nutrition [39]. We evaluated the associations between the use of vasoactive–inotropic medications by obtaining the VIS and calculating enteral nutrition support. Like our findings on pSOFA score trends, enteral caloric adequacy and enteral protein adequacy from day 1 to day 3 were less than 1% for calories and protein when the VIS remained unchanged; in contrast, the adequacy values for calories and protein remained 0 when the VIS trend was up or down. Other studies have evaluated the safety of enteral nutrition while providing vasoactive support. One retrospective study of 399 critically ill children by Panchal et al. evaluated the safety of enteral feeding in children receiving vasoactive agents and concluded that receiving vasoactive–inotropic support is not a contraindication of enteral feeding, as there were no reported adverse gastrointestinal effects. The authors found that children with the highest severity of illness were less likely to be fed enterally [14]. Although the provision of enteral nutrition support is recommended when vasoactive support is stable, our findings indicate that we did not provide adequate enteral nutrition support in this cohort during the first 3 days of admission to a pediatric intensive care unit [5].

The limitations of our study include the following: (1) it is a single-center retrospective study that reflects nutrition support practices from our institution from the study period, and it is possible that current nutrition support practices at our institution have changed after the most recent ASPEN guidelines from 2017; (2) regarding the high prevalence of organ dysfunction, it might be plausible that current and timely interventions for the care of children admitted to the pediatric intensive care unit had decreased the prevalence of organ dysfunction, therefore decreasing the strength of the associations found in this study; and, (3) finally, the most important limitation of our study is the fact that our database was collected almost 10 years ago.

## 5. Conclusions

In summary, our findings indicate that for moderately to severely ill children with organ dysfunction, the use of parenteral nutrition combined with enteral nutrition was needed to reach 60% goal adequacy based on dietitians’ prescriptions and recommended goals from the ASPEN/SCCM guidelines for both calories and protein. Patients with an uptrending pSOFA score on day 3 were associated with an almost insignificant calorie and protein adequacy compared to patients with unchanged or downtrending pSOFA scores. Similarly, patients with a high VIS had zero adequacy for enteral calories and protein, compared to those with a low VIS on day 3, and upward changes in the VIS on day 3 were also associated with zero enteral adequacy compared to children with an unchanged VIS. The evaluation of organ dysfunction and nutritional status upon admission to a pediatric intensive care unit might be useful in choosing the appropriate nutrition support route with which to achieve goal adequacy. Based on these results, we should aim to reach a higher proportion of patients reaching more than 60% adequacy for calories and protein during the first week of admission. These goals can be achieved by the prompt identification of the children who are eligible for early enteral nutrition support, and, if a patient cannot tolerate enteral nutrition, the timely initiation of parenteral nutrition.

## Figures and Tables

**Table 1:** Patient characteristics.

All PICU Admissions	*n* = 1283
Age (years)	2.83 (0.62–10)
Weight (kg)	13.4 (7.3–31.6)
Female/male	554/729
BMI/A z-score	0.11 ± 1.90 (SD)
Prevalence of acute malnutrition, *n* (%)	135 (10.5)
Prevalence of normal nutritional status, *n* (%)	974 (75.9)
Prevalence of obesity, *n* (%)	174 (13.6)
PIM2, ROM (%)	3.1 (0.87–4.5)
pSOFA score day 1	5 (4–7)
Prevalence of organ dysfunction day 1, *n* (%)	843 (65.7)
pSOFA score day 3	5 (3–7)
Prevalence of organ dysfunction day 3, *n* (%)	729 (56.8)
Mechanical ventilation upon admission, *n* (%)	941 (73.3)
Mechanical ventilation (hours)	143 (80–249)
PICU LOS (days)	6.7 (4.4–11)
Hospital LOS (days)	18 (10–33)
Mortality, *n* (%)	89 (6.94)

Continuous variables are represented as medians with interquartile ranges (25–75th) and average ± standard deviation (SD). Categorical variables are expressed as numbers and percentages. kg: kilograms; SD: standard deviation; PIM2: Pediatric Index of Mortality 2; ROM: risk of mortality; BMI/A: body mass index for age; pSOFA: Pediatric Sequential Organ Failure Assessment; and PICU LOS: pediatric intensive care unit length of stay.

**Table 2:** Nutrition support cohort characteristics.

Nutrition Support Cohort	*n* = 164
Age (years)	11.72 (7.73–15.4)
Weight (kg)	36.8 (23.3–55.3)
Female/male	74/90
BMI/A z-score	0.30 ± 1.65 (SD)
Prevalence of acute malnutrition, *n* (%)	13 (7.9)
Prevalence of normal nutritional status, *n* (%)	129 (78.7)
Prevalence of obesity, *n* (%)	22 (13.4)
PIM2, ROM (%)	3.71 (2.83–6.35)
pSOFA score day 1	7.5 (5–10)
Prevalence of organ dysfunction day 1, *n* (%)	130 (79.3)
pSOFA score day 3	7 (5–9)
Prevalence of organ dysfunction day 3, *n* (%)	129 (78.7)
Vasoactive–inotropic score day 1	0 (0–10)
Vasoactive–inotropic score day 3	0 (0–4.8)
Mechanical ventilation on admission, *n* (%)	131 (79.9)
Mechanical ventilation (hours)	223 (157–392)
PICU LOS (days)	11.93 (9.4–20)
Hospital LOS (days)	29.5 (19–53)
Mortality, *n* (%)	20 (12.2)

Continuous variables are represented as medians with interquartile ranges (25–75th) and average ± standard deviation (SD). Categorical variables are expressed as numbers and percentages. kg: kilograms; SD: standard deviation; PIM2: Pediatric Index of Mortality 2; ROM: risk of mortality; BMI/A: body mass index for age; pSOFA: Pediatric Sequential Organ Failure Assessment; and PICU LOS: pediatric intensive care unit length of stay.

**Table 3:** Caloric and protein intake and adequacy.

*n* = 164	Caloric Intake (kcal/kg/day)		Caloric Adequacy (%)		Protein Intake (g/kg/day)		Protein Adequacy (%)	
	Total	Enteral	Total	Enteral	Total	Enteral	Total	Enteral
Day 1	4 ± 11 *	2 ± 8 *	0 (0–0)	0 (0–0)	0.18 ± 0.48 *	0.06 ± 0.23 *	0 (0–0)	0 (0–0)
Day 3	15 ± 19 *	7 ± 16 *	19 (0–60)	0 (0–15)	0.64 ± 0.81 *	0.25 ± 0.55 *	16 (0–70)	0 (0–13)
Day 5	28 ± 22 *	12 ± 20 *	65 (26–100)	2 (0–44)	1.22 ± 0.95 *	0.44 ± 0.69 *	69 (25–105)	2 (0–46)
Day 7	31 ± 22 *	15 ± 22 *	74 (42–104)	6 (0–71)	1.46 ± 1.04 *	0.60 ± 0.85 *	83 (45–124)	6 (0–64)
	Caloric prescription by a dietitian: 44 ± 17 kcal/kg/day				Protein prescription by a dietitian: 1.70 ± 0.42 g/kg/day			

Values are mean ± SD and median (IQR, 25–75th). * *p* < 0.0001 by a paired *t*-test comparing prescription vs. intake. Adequacy was calculated as [(intake/dietitian prescription) × 100].

**Table 4:** Total caloric and protein intake based on organ dysfunction status.

*n* = 164	Calories (kcal/kg/day)		Protein (g/kg/day)	
	Day 1	Day 3	Day 1	Day 3
Organ dysfunction	0 (0–0)	9.3 (0–25.1)	0 (0–0)	0.30 (0–1.27)
No organ dysfunction	0 (0–0)	5.5 (0–19.4)	0 (0–0)	0.24 (0–0.72)

Values are median (IQR). Organ dysfunction was defined as a Pediatric Sequential Organ Failure Assessment score > 5. Analysis via the Mann–Whitney U test comparing the intake of patients with OD vs. the intake of patients without OD; *p* = 0.9124 and *p* = 0.5373 for calories on days 1 and 3, respectively; and *p* = 0.9161 and *p* = 0.3146 for protein on days 1 and 3, respectively.

**Table 5:** Average enteral caloric and protein adequacy values for days 1–3 and vasoactive–inotropic score (VIS) trend.

*n* = 164	Enteral Caloric Adequacy (%)	Enteral Protein Adequacy (%)
VIS trending upward (*n* = 26)	0 (0–0)	0 (0–0)
VIS trending downward (*n* = 46)	0 (0–0)	0 (0–0)
VIS unchanged (*n* = 92)	0.56 (0–25) *	0.48 (0–26) **

Values are median (IQR). Analysis via the Kruskal–Wallis test. *p*-value significant if < 0.0167 for the three-group comparison; * *p* = 0.0012; ** *p* = 0.0010 for enteral caloric and protein adequacy comparison, respectively. Adequacy was calculated as [(intake/prescription) × 100].

## Data Availability

Data are contained within the article.
